# Public Awareness of Sepsis Compared to Acute Myocardial Infarction and Stroke in Jeddah, Saudi Arabia: Questionnaire Study

**DOI:** 10.2196/16195

**Published:** 2020-06-15

**Authors:** Nourah Al-Orainan, Adel Mohamed EL-Shabasy, Khawlah Alamin Al-Shanqiti, Rawan Awad Al-Harbi, Hadeel Rajeh Alnashri, Raghad Ahmed Rezqallah, Alanoud Abdallah Mirghani

**Affiliations:** 1 Department of Medicine King Abdulaziz University Jeddah Saudi Arabia; 2 Department of Anesthesiology and Critical Care King Abdulaziz University Jeddah Saudi Arabia

**Keywords:** sepsis, public awareness, survey

## Abstract

**Background:**

Sepsis is a state of organ dysfunction caused by an impaired host response to infection. It is one of the leading causes of death globally. Sepsis, acute myocardial infarction (AMI), and stroke share the primary management requirement of rapid intervention. This could be achieved through early presentation to the hospital, which demands previous knowledge of the disease to ensure better outcomes.

**Objective:**

Our study aimed to assess the level of public awareness of sepsis compared with AMI and stroke.

**Methods:**

This was a cross-sectional survey study performed in June and July 2018, with 1354 participants from Jeddah, Saudi Arabia, aged ≥18 years. Data entry was performed using Microsoft Excel and statistical analysis including chi-square tests and multilogistic regression was performed using SPSS software.

**Results:**

A total of 1354 participants were included. Only 56.72% (768/1354) had heard of the term “sepsis” and 48.44% (372/768) of these participants were able to correctly identify it. In addition, 88.33% (1196/1354) had heard the term “myocardial infarction” and 64.63% (773/1196) knew the correct definition of that condition. Stroke was recognized by 81.46% (1103/1354) of participants and 59.20% (653/1103) of these participants correctly identified the condition. The difference between those who had heard of these diseases and those who knew the correct definition significantly differed from the values for awareness of sepsis and its definition.

**Conclusions:**

We found that public awareness and knowledge of sepsis are poor amongst the population of Jeddah compared with the awareness and knowledge of AMI and stroke. This lack of knowledge may pose a serious obstruction to the prompt management needed to limit fatal outcomes.

## Introduction

Sepsis is defined as the state of organ dysfunction caused by an impaired host response to infection [1]. It affects up to 30 million people globally every year, potentially leading to 6 million deaths [2]. It is also one of the leading causes of death in critically ill patients, with a mortality rate of 30%-40% [3]. Although epidemiological studies on sepsis are limited in Saudi Arabia, a study conducted in Buraidah Central Hospital, Qassim, revealed that 16% of all patients in intensive care had sepsis, and 40.3% of those cases were fatal [4].

In addition to the high mortality rate, survivors of an initial sepsis episode still face a significant risk of subsequent infections during the following year, posing a threat to their lives despite surviving an acute course [5]. They also experience a substantial reduction in long-term health-related quality of life [6].

The prime components of sepsis management are the rapid restoration of tissue perfusion with intravenous fluid and the suitable control of the infection source. This includes administration of proper antibiotics, drainage of infected fluids, and debridement of infected soft tissues [7].

Amongst the many steps required to manage sepsis, early administration of antibiotics has a significant impact on patient mortality. A retrospective study of 17,000 patients diagnosed with sepsis or septic shock found that a delay in the administration of antimicrobials beyond the first hour postdiagnosis drastically increased mortality [8]. A cohort study of 35,000 patients in the emergency department showed similar results [9]. The 2018 update from the Surviving Sepsis Campaign, which aims to reduce mortality through an evidence-based approach, recommends that management and resuscitation of sepsis cases must be started immediately as part of the 1-hour bundle [10].

In addition to sepsis, there are several other conditions that require rapid intervention, such as stroke and acute myocardial infarction (AMI).

The World Health Organization defines stroke as the rapid development of “clinical signs of focal disturbance of cerebral function, lasting more than 24 h or leading to death with no apparent cause other than that of vascular origin.” Recent epidemiological data show that 13.7 million incidences of stroke annually, 5.5 million of which are fatal [11]. In 2013, stroke was the second and third most common cause of death and disability, respectively, worldwide [12].

AMI is defined as the death of cardiac myocytes due to prolonged ischemia [13]. In 2005, coronary heart disease accounted for 7.6 million deaths, with AMI as one of the primary manifestations [12].

Although it remains one of the leading global causes of mortality [12], the treatment of AMI has undergone a dramatic change over the past decade, significantly reducing the mortality rate [14,15]. Similar changes in the treatment of sepsis have resulted in similar success in management; however, mortality rates have failed to decline as substantially as they have for AMI. This discrepancy in treatment outcomes suggests a need to assess other factors [16].

Since timely intervention is crucial for the management of these conditions, a delay in treatment could be one of the factors contributing to the mortality rate.

Many factors can prevent early treatment, some of which are institution-related, such as the availability of resources and doctors’ level of training. Other factors are patient-related, including late presentation to the hospital, which in turn may be attributed to a lack of awareness [17]. For this reason, several studies have been conducted to assess levels of public awareness.

One of the largest studies was “An international survey: Public awareness and perception of sepsis,” performed using structured telephone interviews of 6021 participants from Europe and America. Results indicated that public awareness of sepsis is low [18]. Similarly, a research study involving 1001 Swedish residents concluded that public knowledge of sepsis is low [19]. Other authors attempted to compare awareness of sepsis with that of other severe conditions, such as a Singapore study that compared knowledge of sepsis with knowledge of stroke and found that knowledge of both conditions was insufficient, but more evident with sepsis [20]. Awareness of sepsis and stroke was studied along with AMI awareness in Korea via web- and paper-based surveys of 1081 individuals. When the three conditions were compared, people were least informed about sepsis [21].

Although some studies have compared sepsis awareness with awareness of AMI and stroke, only a few studies investigated all three conditions in the same study, and no such study has been performed in Saudi Arabia.

Our research aimed to compare the level of public awareness of sepsis with that of AMI and stroke in order to plan proper interventions and improve outcomes.

## Methods

This study was approved by the Institutional Review Board of King Abdulaziz University Hospital (KAUH). This was a cross-sectional study conducted in KAUH, Saudi Arabia, Jeddah. A convenience sample of 1354 participants was calculated to represent the population of Jeddah, which is 3.4 million people, according to the national municipality, with a confidence interval of 99% and a margin of error of 3.5.

Inclusion criteria were resident of Jeddah and age ≥18 years. Data were collected through electronic self-administered questionnaires. In order to mitigate bias, participants were approached on different sites, including KAUH, shopping centers, and mosques in various regions of Jeddah. Data were also collected during different time periods (day and evening shifts) over the course of a month (June 24 to July 24, 2018).

The questionnaire used in this study was previously used in the “Awareness and knowledge of sepsis in the general Korean population” study [21], which measured knowledge of the 3 conditions and consisted of three sections. The first section included 4 questions regarding the awareness and knowledge of sepsis. The second included 2 questions about AMI, and the third asked 2 questions about stroke. The last question determines knowledge of disease impact by asking participants to compare the mortality of sepsis with AMI, stroke, and other presentations including, cardiac arrest, Trauma, Lung cancer, colorectal cancer, stomach cancer.

A section on the most important risk factors and symptoms of each disease in addition to an informed consent section was added.

The questionnaire was forward translated into Arabic and then backward translated into English by native speakers, and the two English copies were compared to ensure meaning compatibility. Once this was achieved, a pilot of 135 people answered the questionnaire to ensure no further adjustment was required, and the final version was distributed among the target sample.

Participants were asked to choose the correct risk factors and symptoms for each condition. With the exception of demographic data, all items called for multiple-choice responses.

Data were collected and entered in Microsoft Excel (Microsoft Corporation), followed by statistical analysis using Statistical Package for the Social Sciences (SPSS; version 21; IBM Corporation) to perform chi-square tests and multiple logistic regression analysis; *P*<.05 was considered significant.

## Results

Our research aimed to assess the level of public awareness of sepsis compared with AMI and stroke in order to plan proper interventions and improve outcomes.

### Demographics

A total of 1354 participants aged ≥18 years were included, with a mean age of 30.41 (SD 11.2) years. Of the total sample, 951/1354 (70.2%) were female and 403/1354 (29.8%) were male (Table 1).

**Table 1:** Participant demographics (N=1354).

Demographic			Value, n (%)
**Age (years)**			
	≤19	14.99% (203/1354)	
	20-29	40.84% (553/1354)	
	30-39	23.48% (318/1354)	
	40-49	11.89% (16/1354)	
	50-59	6.64% (90/1354)	
	60-69	1.84% (25/1354)	
	70-90	0.29% (4/1354)	
**Sex**			
	Male	29.76% (403/1354)	
	Female	70.23% (951/1354)	
**Education**			
	Elementary or less	1.69% (231/354)	
	Middle school graduate	4.35% (59/1354)	
	High school graduate	28.50% (386/1354)	
	University or college students	59.89% (811/1354)	
	Postgraduate	5.53% (75/1354)	

### Awareness of Sepsis Versus Acute Myocardial Infarction and Stroke

Of the 1354 participants, 56.72% (768/1354) had heard of the term “sepsis”; however, only 48.43% (372/768) of these knew the correct definition, comprising 27.47% (372/1354) of the overall study population. The term “myocardial infarction” was familiar to 88.33% (1196/1354) participants, and 64.63% (773/1196) of them knew the correct definition. The term stroke was known to 81.46% (1103/1354) participants, and 59.20% (653/1103) of them knew the correct definition (Table 2).

Chi-square test suggested a significant difference between the numbers of individuals who had heard of the term “sepsis” and those who had only heard of AMI and stroke (*P*<.001 in both cases).

When asked about the correct definition of sepsis, 48.44% (372/768) chose “severe systemic inflammatory response to infection,” 24.61% (189/768) chose “I’m not sure,” 8.33% (64/768) chose “systemic poisoning as a result of ingestion of expired food,” 7.81% (60/768) chose “severe allergic reaction,” 2.47% (19/768) chose “systemic poisoning by raw fish or milk,” and 2.08% (16/768) chose “other.” Knowledge of the risk factors and symptoms of sepsis are listed in (Table 3).

When asked about AMI, 64.63% (773/1196) of respondents chose “death of heart cells or tissues due to occlusion of heart blood vessels,” 12.12% (145/1196) chose “irregular heartbeat,” 10.87% (130/1196) chose “not sure,” 5.51% (66/196) chose “slow heart beats,” 2.09% (25/1196) chose “inflammation of heart muscle,” and 0.17% (2/1196) chose “other.” Knowledge of the risk factors and symptoms of AMI is described in Table 3.

When asked how to define stroke, 59.20% (653/1103) of participants answered “brain dysfunction due to occlusion or rupture of blood vessel,” 22.57% (249/1103) chose “traumatic injury to brain,” 11.70% (129/1103) chose “not sure,” 3.17% (35/1103) chose “inflammation to brain tissue,” 3.26% (36/1103) chose “type of brain tumour,” and 0.09 % (1/1103) chose “other.” Survey results regarding the risk factors and symptoms of stroke are described in Table 3.

A significant difference was found in the likelihood of respondents selecting the correct definition of sepsis versus that of AMI and sepsis (*P*<.001).

### Mortality of Sepsis

Participants’ assessment of sepsis mortality was underestimated compared with other diseases. When asked to compare sepsis mortality rates with those of other disease, three conditions were thought to produce higher mortality rates than sepsis. cardiac arrest was thought to cause more deaths by 87. 89% (1190/1354) of respondents. AMI came second 72.82 % (986/1354), followed by stroke 71.20% (964/1354), and lung cancer 57.68 % (781/1354) . Sepsis was thought to be more fatal than several other illnesses including trauma 66.99% (907/1354), stomach cancer 55.76% (755/1354), and colorectal cancer 54.87% (743/1354).

### Source of Information

Participants’ sources of information varied, yet media/internet was the most common source at 38% (514/1354) followed by friends/family at 20.8% (295/1354), school at 16% (217/1354), hospital or medical personnel at 8.1% (110/1354), self or relatives at 7.6% (103/1354), and other at 0.9% (122/1354); 4.8% (650/1354) were not sure.

### Factors Affecting Awareness

A multiple logistic regression analysis was conducted to study factors affecting awareness, and the results revealed that education (college or above) was a predictor of term knowledge (odd ratio 2.787, 95% CI 1.25-6.201, *P*=.012), yet gender and age were not significant.

**Table 2:** Participant knowledge of the terms sepsis, myocardial infarction, and stroke (N=1354).

Number of participants who have heard of the term	Yes, n (%)	No, n (%)	Not sure, n (%)
Those who have heard of the term sepsis	768 (56.72)	500 (36.90)	86 (6.40)
Those who have heard of the term acute myocardial infarction	1196 (88.33)	116 (8.57)	42 (3.10)
Those who have heard of the term stroke	1103 (81.46)	174 (12.85)	77 (5.69)

**Table 3:** Participant knowledge of sepsis, myocardial infarction, and stroke risk factors and symptoms.

Symptoms and risk factors			Correctly answered questions, n (%)	Correctly identified all, n (%)
**Sepsis, n=768**				
	**Risk factors**			12.65
		Low immunity	637 (82.94)	
		Burns/injuries	3637 (47.26)	
		Diabetes	287 (37.36)	
		Tubes/catheters	482 (62.76)	
	**Symptoms**			14.1
		Rapid heartbeat	276 (35.93)	
		Fever	373 (48.57)	
		Difficulty breathing	519 (67.57)	
		Altered mentation	338 (44.0)	
**Myocardial infarction, n=1196**				
	**Risk factors**			47.74
		High cholesterol	968 (80.94)	
		High blood pressure	1037 (86.71)	
		Smoking	1080 (90.30)	
		Obesity	1030 (86.12)	
		Diabetes	716 (59.87)	
	**Symptoms**			33.96
		Difficulty breathing	1021 (85.37)	
		Chest pain	1042 (87.12)	
		Rapid heartbeat	903 (75.50)	
		Arm/Jaw/Back pain	722 (60.37)	
		Sweating	789 (66.07)	
**Stroke, n=1103**				
	**Risk factors**			27.28
		Diabetes	590 (53.49)	
		Obesity	534 (48.41)	
		Smoking	714 (64.73)	
		Hypertension	959 (86.94)	
		High cholesterol	748 (67.82)	
	**Symptoms**			42.24
		Difficulty speaking	908 (82.32)	
		Facial drooping	789 (71.53)	
		Altered mental status	892 (80.87)	
		Weak arm or leg	615 (55.85)	

## Discussion

### Awareness of Sepsis in Jeddah Compared to the World

Our study aimed to assess the level of public awareness of sepsis compared with AMI and stroke.

Sepsis is a serious health concern; if not managed promptly, it could lead to death. Delay in sepsis management could be attributed to a lack of awareness as suggested by research studies such as the Rubulotta international survey [18]. Only a limited number of studies have compared sepsis knowledge with knowledge of other conditions for which treatment time is critical, and none were completed in Saudi Arabia. We distributed a self-filled modified sepsis-awareness questionnaire [21] among 1354 residents of Jeddah and found a significant difference in the level of sepsis knowledge compared to knowledge of stroke and AMI.

Our results also showed that only 56.72% of respondents had heard of sepsis, which was less than the level of awareness found in the Korean population (76.9%) [21] but higher than that in other countries included in the international survey, such as the United Kingdom (14%), Spain (13%), France (4%), Italy (8%), the United States (19%), Germany (52%), and Singapore (0.5%) [18-20]. The differences between our study and previous ones may be attributable to the fact that the Arabic translation of the word “sepsis” is self-explanatory to some extent.

The percentage of people who can identify the correct definition of sepsis is 48.43%, which is slightly higher than the majority of the countries in previous studies, ranging from 4.2% to 47% [18,20,21]. Although our study found higher numbers than previous studies, the overall percentage of those who know the correct definition among the entire population is 27.47%, which is consistent with 27.3% in the Korean community. This reflects how poor sepsis awareness is within our society.

Several factors may influence the level of knowledge in the polled community. For example, most of the participants (65.42%) are well educated (college and above), and those with a college education and above tend to be more knowledgeable (*P*=.012). This is similar to the findings in Singapore, although females in that study were significantly more likely to know the term than they were in this study.

The second part of the questionnaire tested knowledge of AMI. We found that 88.3% of participants had heard of the term, and 64.63% of them knew the correct definition. Much like the Korean population, the difference between knowledge of sepsis and AMI is strongly significant (*P*<.001), although the overall knowledge of AMI in this study was slightly lower than that in the Korean population in which 94.3% have heard of the term and 80.0% identified the correct definition [21].

Similarly, when asked about stroke, 81.46% had heard of the term, and 59.20% of them knew the correct definition. This is again lower than the numbers in Korea (96.9% and 93.1%, respectively) [21]; however, this result is similar to the findings of a study completed in Riyadh, Saudi Arabia, where 87.7% of the population had heard of the term [22]. Despite this difference, knowledge of stroke and its correct definition is still significantly higher than that of sepsis (*P*<.001).

To further evaluate the level of knowledge and identify which aspects of the disease were familiar to the public, we asked participants who had heard of the term “sepsis” to choose the correct risk factors and symptoms of the disease; only 12.65% were able to select all the correct risk factors. The most chosen risk factor was low immunity (82.94%). When asked about symptoms, 14.19% knew all symptoms provided in the questionnaire, and the most frequently chosen symptom was difficulty breathing (67.57%). When asked to choose risk factors for AMI, 47.74% correctly identified all that applied. Of those who had heard of the term, 90.30% chose smoking as a risk factor. When asked about the symptoms of AMI, 33.96% correctly identified all symptoms that applied, and the most frequently chosen symptom was chest pain (87.12%).

When asked about stroke, 27.28% of participants who had heard of the term could correctly identify all risk factors, with the most common 86.94% being hypertension. When asked to identify the symptoms of stroke, 42.24% correctly identified all relevant symptoms, with the most common 82.32% being difficulty speaking.

To the best of our knowledge, no research has been conducted to characterize public knowledge of risk factors and symptoms of sepsis. In our study, knowledge of risk factors and symptoms varied, yet sepsis knowledge was the lowest of the three conditions.

In addition to the poor awareness of sepsis, its mortality is underestimated; people of Jeddah placed it after cardiac arrest, AMI, stroke, and lung cancer. This may correlate with the fact that sepsis symptoms are vague and unspecific, often intersecting with the features of other diseases, so deaths may be misattributed to illnesses other than sepsis [23], particularly if knowledge of these symptoms is lacking. This leads to an underestimation of its seriousness and a misrepresentation of the actual mortality rate.

Findings suggest knowledge of stroke and AMI is significantly higher than knowledge of sepsis. One possible reason is that both have characterized signs and symptoms that are familiar to the public and have been promoted through various campaigns. For example, The Saudi Stroke Association, which was established in 2006, has been raising public awareness as part of its goal to reduce poor outcomes [24].

Similar efforts have been made to enhance knowledge of cardiovascular diseases [25,26]. Only a few such attempts have been made to increase awareness of sepsis. The National Sepsis Reduction Campaign launched in Riyadh in April 2018 is one such example.

Previous studies showed that this high awareness of AMI and stroke led to reductions in late presentation to the hospital [27,28]. Sepsis needs rapid management as well; therefore, more public education for sepsis is necessary to improve recognition of the seriousness of the disease and reduce delays in presentation to the hospital.

In our study, internet/media was cited as the predominant source of information (38%), suggesting that upcoming awareness campaigns should use this format to improve reach and efficacy.

Our study has several limitations. Our questionnaire was multiple choice, which could yield higher estimates of correct answers due to chance or random selection. Most of the sample group was also well educated. Although this reflects the overall educational status of Jeddah’s population, this does not necessarily represent the entire country’s population. These two points could have positively biased the results and limited their generalizability.

### Conclusion

Our study aimed to assess the level of public awareness of sepsis and compared it with that of AMI and stroke. We found that public awareness and knowledge about sepsis is inadequate within the population of Jeddah compared with that of AMI and stroke. This may obstruct the prompt management needed to limit the mortality of sepsis.

More attempts to raise awareness are crucial. Coordinated efforts should be made to place well defined, applicable, and time-framed strategies to reach this goal. International Sepsis Day constitutes a valuable opportunity to improve the reach of societal awareness campaigns. We suggest using media and the internet as a platform to involve the public and deliver important information.

In order to reach older members of the community and those who have no access to the internet, we recommend targeting visitors of primary health care centers, chronic disease clinics, and hospitals, each of which particularly serves the population most at risk. Education could be distributed through posters, banners, and verbal counseling by physicians and regulated efforts must be implemented to train health care providers on appropriate methods of patient education.

### Future Studies

Future studies should use a validated questionnaire to assess participants’ awareness of sepsis, stroke, and AMI more in depth and question their knowledge of not only the terms and common symptoms but also the proper response when symptoms present and are recognized. We recommend the use of interventional studies to assess the impact of efforts made to improve public awareness of sepsis.

## Abbreviations

AMI: acute myocardial infarction
KAUH: King Abdulaziz University Hospital
